# Domain knowledge-assisted multi-objective evolutionary algorithm for channel selection in brain-computer interface systems

**DOI:** 10.3389/fnins.2023.1251968

**Published:** 2023-09-07

**Authors:** Tianyu Liu, An Ye

**Affiliations:** School of Information Engineering, Shanghai Maritime University, Shanghai, China

**Keywords:** channel selection, brain-computer interface systems, multi-objective optimization, two-objective problem model, domain knowledge

## Abstract

**Background:**

For non-invasive brain-computer interface systems (BCIs) with multiple electroencephalogram (EEG) channels, the key factor limiting their convenient application in the real world is how to perform reasonable channel selection while ensuring task accuracy, which can be modeled as a multi-objective optimization problem. Therefore, this paper proposed a two-objective problem model for the channel selection problem and introduced a domain knowledge-assisted multi-objective optimization algorithm (DK-MOEA) to solve the aforementioned problem.

**Methods:**

The multi-objective optimization problem model was designed based on the channel connectivity matrix and comprises two objectives: one is the task accuracy and the other one can sensitively indicate the removal status of channels in BCIs. The proposed DK-MOEA adopted a two-space framework, consisting of the population space and the knowledge space. Furthermore, a knowledge-assisted update operator was introduced to enhance the search efficiency of the population space by leveraging the domain knowledge stored in the knowledge space.

**Results:**

The proposed two-objective problem model and DK-MOEA were tested on a fatigue detection task and four state-of-the-art multi-objective evolutionary algorithms were used for comparison. The experimental results indicated that the proposed algorithm achieved the best results among all the comparative algorithms for most cases by the Wilcoxon rank sum test at a significance level of 0.05. DK-MOEA was also compared with a version without the utilization of domain knowledge and the experimental results validated the effectiveness of the knowledge-assisted mutation operator. Moreover, the comparison between DK-MOEA and a traditional classification algorithm using all channels demonstrated that DK-MOEA can strike the balance between task accuracy and the number of selected channels.

**Conclusion:**

The formulated two-objective optimization model enabled the selection of a minimal number of channels without compromising classification accuracy. The utilization of domain knowledge improved the performance of DK-MOEA. By adopting the proposed two-objective problem model and DK-MOEA, a balance can be achieved between the number of the selected channels and the accuracy of the fatigue detection task. The methods proposed in this paper can reduce the complexity of subsequent data processing and enhance the convenience of practical applications.

## 1. Introduction

Brain-computer interface (BCI) systems establish a connection between the brain and external devices by acquiring brain signals to control external devices (Khan et al., 2020). Therefore, BCIs have provided great convenience for helping paralyzed patients or controlling games. According to the different signal acquisition methods, BCIs can be divided into invasive (Rapeaux and Constandinou, 2021) and non-invasive (Jo and Choi, 2018; Zhuang et al., 2020). In non-invasive BCIs, electroencephalography (EEG) signals are acquired by external sensors with multiple channels (Carneiro et al., 2020; Singh et al., 2021). In EEG systems, the greater number of the channels, the more comprehensive the signal obtained. Lots of researchers use multi-channel EEG signals (usually 32-channel, 62-channel, or more channels' EEG signals of the entire brain) for emotion recognition to improve classification accuracy (Yu and Yu, 2021). However, many EEG channels contain noise or redundancy, which is detrimental to emotion recognition in practice (Wosiak and Dura, 2020; Al-Saegh et al., 2021). Furthermore, the large number of EEG channels makes data acquisition difficult and increases the computational complexity of data processing. Therefore, it is necessary and important to choose appropriate channels in BCIs.

In recent years, many efficient channel selection algorithms have emerged, such as correlation-based methods, machine-learning-based methods, wrapper-based methods, heuristic-searching-based methods, and so on. Correlation-based method (Park and Chung, 2020; Liu T. et al., 2021; Tiwari and Chaturvedi, 2021) goes through the EEG signals obtained from each channel and uses various information-theoretic concepts to evaluate the correlated channels for each feature and to select these channels. So far, some information theoretic concepts, such as normalized mutual information (NMI) (Yang et al., 2021), entropy (Ghembaza and Djebbari, 2022), correlation coefficient (Jin et al., 2019; Moon et al., 2020), and chi-squared statistics (Baig et al., 2020), have been used to assess the correlation of channels. Machine-learning-based methods (Siddiqui et al., 2020) generally select proper channels by training on the features extracted from the obtained EEG signals with the help of classic machine-learning techniques like the neural network. Wrapper-based methods (Liu Q. et al., 2021; Yavandhasani and Ghaderi, 2021) usually adopt predictors to solve the channel selection problem and tune wrappers according to the specific interaction between classifiers and datasets. As an efficient way to solve NP-compete problems, heuristic-searching-based methods have been adopted to solve channel selection problems successfully. Some commonly used heuristic algorithms are genetic algorithms (Moctezuma and Molinas, 2020), particle swarm optimization (Qi et al., 2020), simulated annealing (Yang, 2020), ant colony optimization (Miao et al., 2020), differential evolution (Hajizamani et al., 2020), and so on.

However, most of the above-mentioned algorithms focus on optimizing the numbers of the selected channels. The goal of the channel selection problem is to trade off a balance between reducing the number of channels and improving the accuracy of classification tasks. In this case, multi-objective evolutionary algorithms (MOEAs), which can balance multiple optimization objectives at the same time, have been introduced to solve channel selection problems in recent years. Some classic MOEAs, such as multi-objective evolutionary algorithm based on decomposition (MOEA/D), multi-objective particle swarm optimization (MOPSO), and non-dominated sorting genetic algorithm (NSGA-II) have been successfully applied for channel selection in the task of single modality based BCIs (Al-Qazzaz et al., 2019; Nandy et al., 2019; Baysal et al., 2021; Li et al., 2022). Few of the existing multi-objective channel selection algorithms consider problem domain-related knowledge in the design of key operators. Existing research has proved that knowledge related to the problem domain can help algorithms find high-quality solutions (Luong et al., 2015). This paper proposes a domain knowledge-assisted multi-objective evolutionary algorithm, called DK-MOEA, to solve channel selection problems in BCIs. DK-MOEA contains two spaces, namely knowledge space and population space. In DK-MOEA, the problem domain-related knowledge stored in the knowledge space is adopted to guide the evolution process of the population space.

In BCIs, the processing of EEG signals is also a crucial factor affecting the performance of algorithms. The majority of the traditional studies of EEG signal processing have been conducted on raw data (Mak et al., 2013; Tong et al., 2018; Ganguly and Singla, 2019). Few of these approaches take into account the relationship between different brain regions. Brain connectivity is now actively used in neuroscience research, and the effectiveness of brain connectivity features in identifying emotional states has been demonstrated (Gaur et al., 2021). Therefore, the EEG signal connection information has been adopted for classification tasks in recent years (Chen et al., 2015; Moon et al., 2018, 2020). Studies have shown that using the EEG connectivity matrix between EEG channels, which describes the connectivity information of different EEG channels, can improve the accuracy of classification tasks (Moon et al., 2020). However, the direct use of the EEG connectivity matrix data does not account for the redundant and invalid information in the connectivity matrix. Therefore, this paper adopts a threshold matrix to filter the EEG connectivity matrix and then determine whether a channel can be deleted. In DK-MOEA, The evolution of the threshold matrix is carried out with the help of the knowledge space, which contains the knowledge including the physical distance between channels and the location of the channels. The main contributions of this paper are listed below:

• A two-objective channel selection problem based on channel connectivity matrix has been formulated.
• A two-space framework, which consists of the population space and knowledge space, is introduced. The knowledge space stores the domain-related information, namely the locations of channels and the distance matrix between channels.
• A knowledge-assisted update operator is proposed to guide the evolution process of the population space with the help of the knowledge space.

## 2. Materials and methods

### 2.1. Data acquisition and processing

Nine volunteers, which ranged in age from 21 to 30, were asked to perform a fatigue detection task in an electromagnetically shielded room in the Brain Cognition and Intelligent Computing Laboratory at Tongji University, China. In this task, the volunteers experienced a wake-sleep-wake physiological process after lunch, since most people experience symptoms of fatigue at this time of day. To ensure the validity of the experiment, all volunteers awoke before 8:30 a.m. and were free of alcohol and drugs. The volunteers first lay on the bed with their eyes closed, and then opened their eyes after hearing the instructions in the headset. One volunteer was considered awake if he/she opened his/her eyes within 2 s, otherwise, the volunteer was considered fatigued.

**Figure 1:**
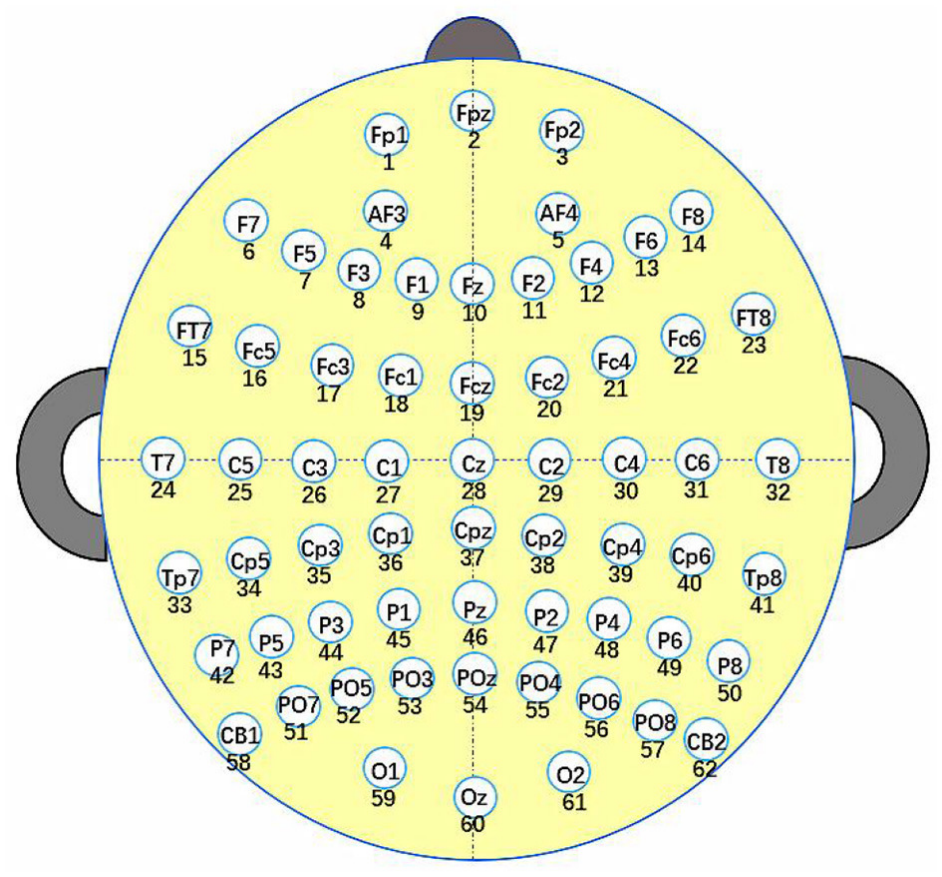
Electrodes positions based on the standard international 10-20 system.

**Figure 2:**
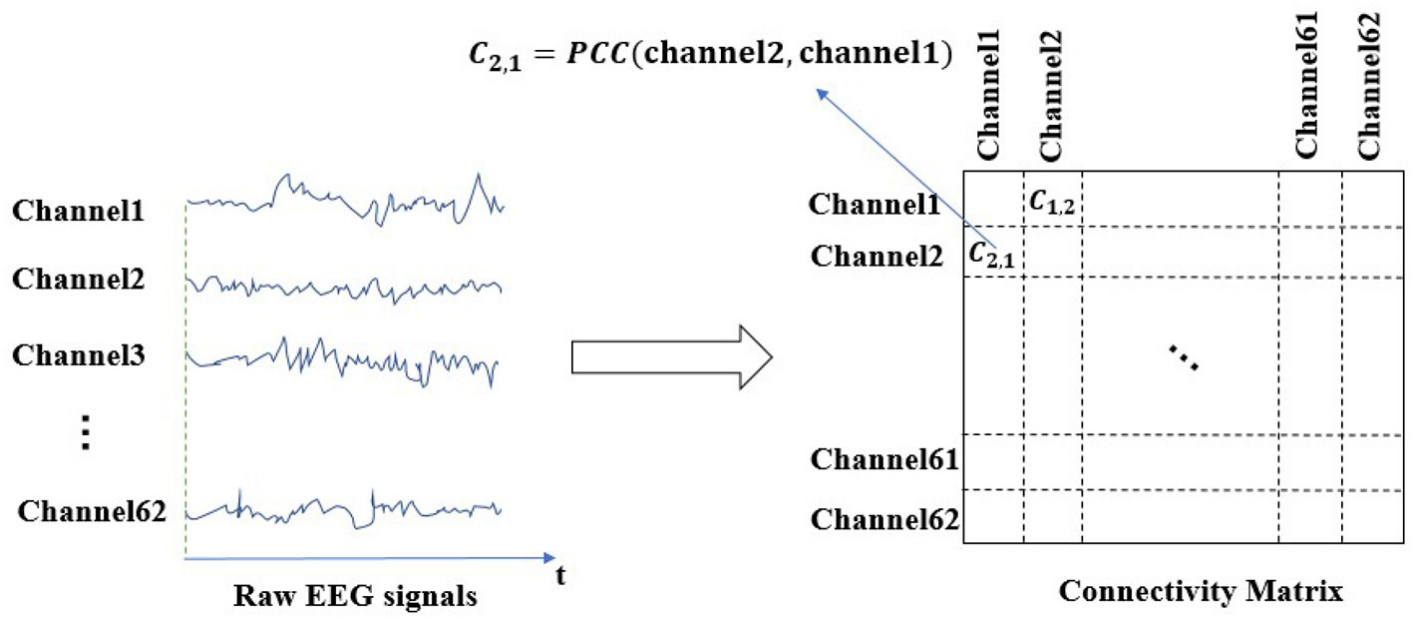
EEG signal processing.

The 62 channels EEG and 2 channels EOG signals were recorded using an ESI-64 channels high-Resolution system (SynAmps2, Neuroscan) (Cao et al., 2010). The 62 electrodes were put following the international 10-20 standard to obtain EEG signals, as shown in Figure 1. The contact impedance of the cortex was calibrated to be less than 5 kΩ. The sampling frequency was 1,000 Hz, which was down-sampled to 250 Hz subsequently for ease of data processing. The recording signal was then filtered between 0 and 40 Hz for further processing. After that, the raw EEG signal is intercepted every 5 s with a sample window and a sliding window of 5 s to convert the analog signal to a digital signal. As shown in Figure 2, the EEG connectivity matrix can be obtained by calculating the correlation coefficient between channels. Recently, The pearson correlation coefficient (PCC) (Pearson, 1895), phase locking value (PLV) (Lachaux et al., 1999), and transfer entropy (TE) (Schreiber, 2000) have been widely adopted to calculate the correlation coefficient in BCIs. PCC measures the linear correlation between two signals. PCC takes values between −1 and 1. PCC = 0 indicates that the corresponding signals are linearly uncorrelated. PCC = −1 and PCC = 1 respectively, represent negative and positive linear relationships between signals, respectively. Suppose $X_{i}=\left\{x_{i}^{1},x_{i}^{2},...,x_{i}^{T}\right\}$ is the EEG signal of the *i*^*th*^ channel, T is the length of the signal, μ_*i*_ *and σ*_*i*_ are the mean and standard deviation of the *i*^*th*^ signal, respectively. The PCC value of signals *X*_*i*_ *and X*_*k*_ can be calculated as shown in Equation (1).


(1) $$\begin{array}{l} PCC(i,k)=\frac{\frac{1}{T}\sum_{t=1}^{T}\left(X_{i}^{t}-\mu_{i})(X_{k}^{t}-\mu_{k}\right)}{\sigma_{i}\sigma_{k}} \end{array}$$

PLV, which can be calculated as Equation (2), describes the phase synchronization between two signals by averaging the absolute phase discrepancies. In Equation (2) , φ_*t*_∈ [0,1] is the phase of the signal at time *t*, *j* is the the imaginary unit.


(2) $$\begin{array}{l} PLV\left(i,k\right)=\frac{1}{T}|\overset{T}{\underset{t=1}{\sum }}e^{j\left(\varphi_{i}^{t}-\varphi_{t}^{t}\right)}| \end{array}$$

TE measures the directed flow of information from signal *X*_*i*_ to signal *X*_*k*_, as shown in Equation (3). In other words, TE describes the advantage of having *X*_*i*_ for predicting *X*_*k*_. TE = 0 indicates that no causal relationship exists between the two-time series.


(3) $$\begin{array}{l} TE\left(i\to k\right)=\frac{1}{T-1}\overset{T-1}{\underset{t=1}{\sum }}p\left(X_{i}^{t},X_{k}^{t},X_{k}^{t+1}\right)\log\frac{p\left(X_{k}^{t+1}|X_{k}^{t},X_{k}^{t}\right)}{p\left(X_{k}^{t+1}|X_{k}^{t}\right)} \end{array}$$

Studies have shown TE performs relatively worse than PCC and PLV, while PCC and PLV have comparable performances (Moon et al., 2020). Compared with PLV, the calculation of PCC is simpler and faster. Therefore, the EEG connectivity matrix was obtained by calculating the PCC values between channels in this paper. It can be observed in Figure 2, *c*_21_ gives the PCC value of channel 2 and channel 1.

### 2.2. Two-objective channel selection problem formulation

This paper aims to reduce the number of the selected channels as much as possible to achieve a balance between the number of selected channels and the classification accuracy using the connectivity matrix.

**Figure 3:**
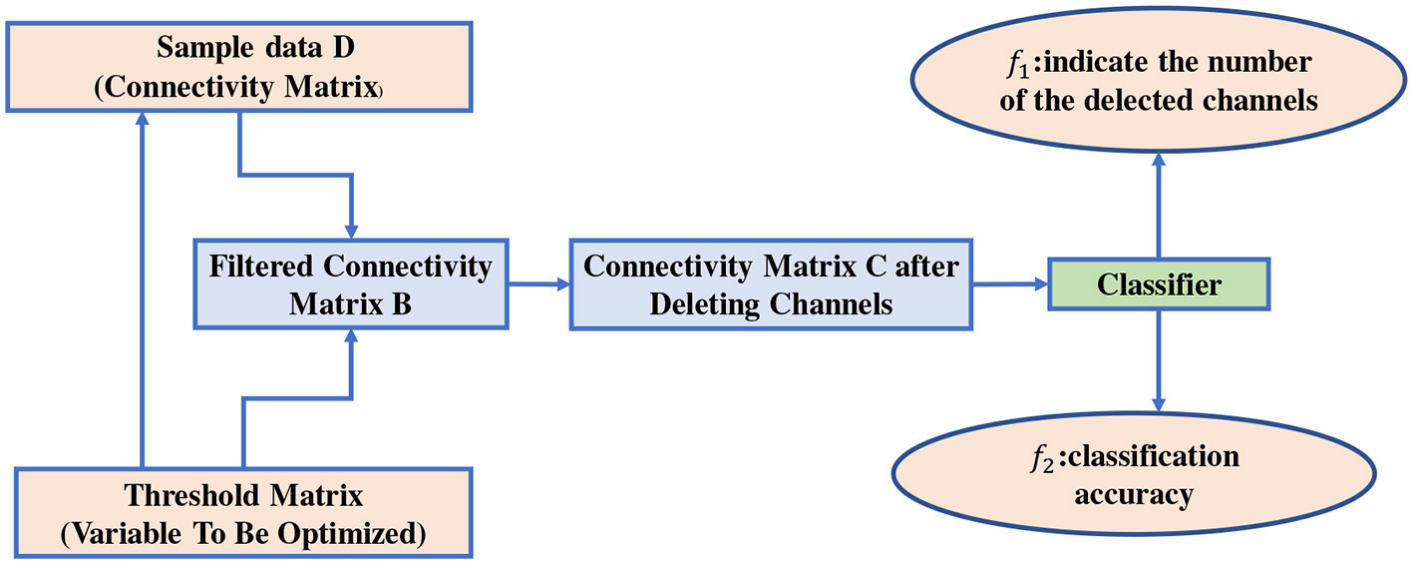
Channel selection problem.

In this paper, the main framework of the channel selection problem is shown in Figure 3. As shown in Figure 3, the core idea is to filter the connectivity matrices of all samples *D* = {*D*_1_, …, *D*_*n*_} with the help of the threshold matrix *X*, each individual, according to the concept of population evolution, has an independent threshold matrix. Then obtain the channels that can be deleted by the filtered connectivity matrix *B* = {*B*_1_, …, *B*_*n*_}. The size of threshold matrix is the same as that of the connectivity matrix and *n* is the number of samples, the *n* is determined by the amount of raw EEG signals data, the sample window, and sliding window sizes as shown in Section 2.1. The final classification accuracy is obtained according to the connectivity matrix *C* = {*C*_1_, …, *C*_*n*_} after deleting part of the channels. To balance the number of the selected channels and the classification accuracy, the channel selection problem is formulated as a two-objective problem, as shown in Equation (4).

In Equation (4), *f*_1_(*X*) is the classification accuracy and *f*_2_(*X*) is the degree to which *X* filters the connectivity matrices of all samples from *D* to *C*, and they are conflicting optimization objectives. Any classifier can be used to obtain classification results [i.e., *f*_1_(*X*)] and the classic SVM is adopted in this paper. The grid search method and the five-fold cross-validation are adopted to obtain the classification accuracy of SVM. Specifically, the grid search method first determines the parameter grid of the possible values for each hyperparameter to obtain all combinations of the possible values for all hyperparameters. Then, the five-fold cross-validation method is used for each set of hyperparameter combinations to select the hyperparameter combination with the best performance for SVM. This paper emphasizes on the investigation of reducing the selected channels while trade-off the classification accuracy by evolving the threshold matrix *X* to filter the EEG connectivity matrices. Therefore, the classifier is not the focus of this paper. In *f*_2_, $\frac{zero\left(C\right)}{N_{C}}$ represents the proportion of the zero elements in *C* = {*C*_1_, …, *C*_*n*_}, where *zero*(*C*) is the number of zero elements and *N*_*C*_ is the total number of elements in *C* = {*C*_1_, …, *C*_*n*_}. $\frac{N_{Cchannel}}{N_{Channel}}$ is the average proportion of the deleted channels, *N*_*Cchannel*_ is the average of the deleted channels for all samples and *N*_*Channel*_ is the total number of channels. The reason for not using $\frac{N_{Cchannel}}{N_{Channel}}$ as *f*_1_ directly is that $\frac{N_{Cchannel}}{N_{Channel}}$ may remain unchanged for a long time and cannot provide sufficient guidance for the evolution process.


(4) $$\begin{array}{l} Maximum\;F(X)=(f_{1}(X),f_{2}(X))\\ f_{1}(X)=Classifier(D,X)\\ f_{2}(X)=0.5*\frac{zero(C)}{N_{C}}+0.5*\frac{N_{Cchannel}}{N_{channel}} \end{array}$$

(5) $$\mu_{t}=\{\begin{array}{ll} m-1 & t=10\\ \mu_{\left(t-1\right)}-1 & mod(t,5)\text{​ }=\text{ ​}0\;and\;t>10 \end{array}$$

The detailed procedure of how to get *B* and *C* is shown in Algorithm 1. In Algorithm 1, *B*_*k*_ (*k*∈1, …, *n*) can be obtained by comparing the values in *D*_*k*_ (*k*∈1, …, *n*) with the corresponding values in *X* = {*x*_1_, *x*_2_, …, *x*_γ_}, respectively. Therefore, *B*_*k*_ is the connectivity matrix after filtering for the *k*^*th*^ sample of *D*. If *B*_*k*_(*i, j*) = 0 (*i*≠*j*), then the correlation coefficient between the *i*^*th*^ channel and the *j*^*th*^ channel is 0 (Lines 2–7 in Algorithm 1). For each column *j* in *B*_*k*_, count the number of zero values in this column and denote it as *z*_*j*_. If *z*_*j*_>μ, then the *j*^*th*^ channel is considered to be deleted for the *k*^*th*^ sample (Lines 8–12 in Algorithm 1). In this paper, μ, which can be obtained according to Equation (6), is set to be constantly changing as the algorithm runs rather than a fixed value. In Equation (6), *m* is the number of channels and t is the number of generations. In the early stage of the algorithm, μ is set to a relatively large value to avoid deleting too many channels prematurely. In the late stage of the algorithm, μ is set to be a relatively small value to help increase the population diversity and improve the search ability. Each stage's non-dominated solutions are stored for subsequent data processing. If the *j*^*th*^ channel can be deleted for all samples, then *C*_1_, …, *C*_*n*_ can be obtained by replacing all elements in the *j*^*th*^ row and column of *B*_1_, …, *B*_*n*_ with 0, respectively (Lines 14-19 in Algorithm 1).

**Algorithm 1:**
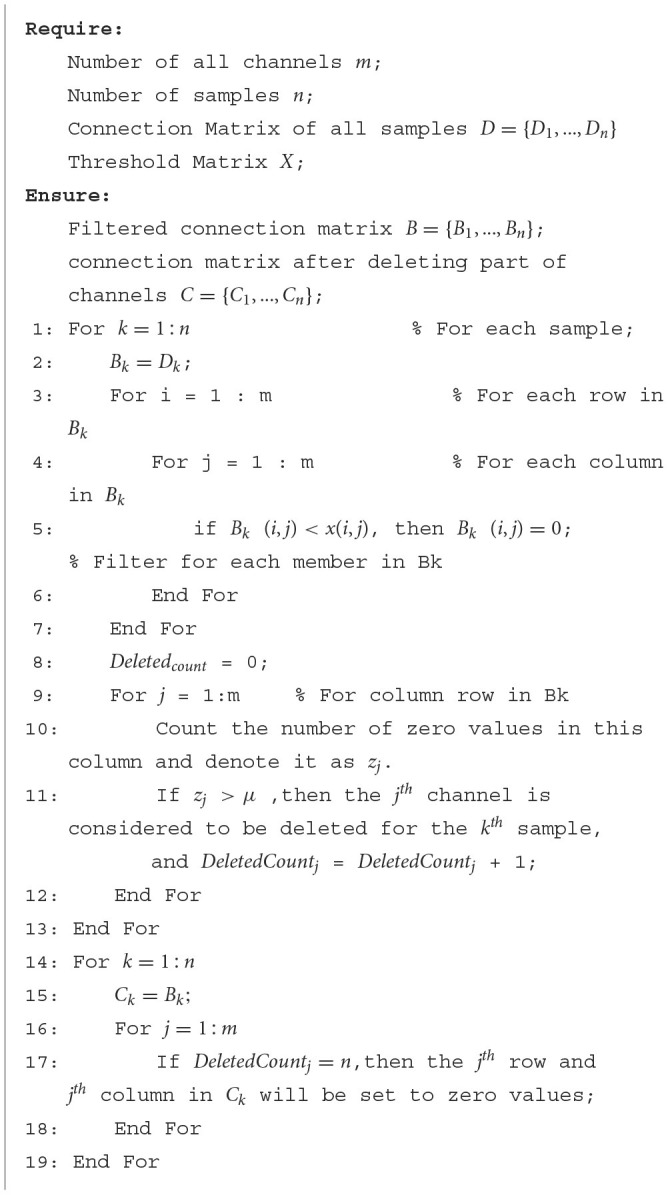
The detailed procedure of obtaining the connection matrix after filtering and deleting.

### 2.3. Proposed DK-MOEA

For ease of understanding, this section starts with the basic knowledge of multi-objective optimization problems (MOPs) and MOEAs, then the general framework and the key operators in the proposed DK-MOEA are given.

#### 2.3.1. Background on MOPs and MOEAs

Many real word problems have multiple conflicting objectives, which are called multi-objective optimization problems (MOPs) (Deb, 2014). Taking the maximization problem as an example, a unconstrained MOP can be expressed as shown in Equation (6). Where *X* = {*x*_1_, …, *x*_*n*_} is a candidate solution and Ω is the search space, *n* and *M* are the dimensions of the search space and objective space, respectively.


(6) $$\begin{array}{l} \begin{array}{cc} Maximum\; & F\left(X\right)={\left(f_{1}\left(X\right),f_{2}\left(X\right),...,f_{M}\left(X\right)\right)}^{T}\\ x\in \Omega \end{array} \end{array}$$

For MOPs, it is usually impossible to find an optimal solution that can optimize all objectives simultaneously. Suppose a maximized MOP, solution *x* dominate *y* (*x*≻*y*), if and only if *x* is not worse than *y* for all objectives and *x* is better than for at least one objective. In this case, *x* and *y* have a Pareto dominance relationship. If solution x^*^ cannot be dominated by any other solutions, then x^*^ is regarded as a Pareto-optimal solution. For MOPs, algorithms need to find a Pareto-optimal set, which contains a set of Pareto-optimal solutions (non-dominated solutions), rather than a single optimal solution. MOEAs have been widely adopted in solving MOPs since they can obtain a set of solutions in one run. The existing MOEAs can be mainly divided into the following categories: Pareto-dominance-based MOEAs, indicator-based MOEAs, and decomposition-based MOEAs. The basic idea of the Pareto-dominance-based MOEAs is to evolve the individual population with the help of Pareto-dominance-based strategies, such as selection and fitness assignment. Typical algorithms are NSGA-II (Deb et al., 2000), NPGA (Erickson et al., 2002), SPEA2 (Zitzler et al., 2001), MGAMOO (Coello Coello and Pulido, 2001), and so on. Pareto-dominance-based MOEAs have shown good performance in solving a variety of MOPs. However, researchers have found that the performance of this type of MOEAs may degrade when dealing with some MOPs, such as MOPs with many objectives (Falcón-Cardona et al., 2019). This is because the selection pressure decreases dramatically as the number of targets increases in Pareto-dominance-based MOEAs. Indicator-based MOEAs use different performance metrics to guide the search process of algorithms. Various metrics have been proposed, including Inverted Generational Distance (IGD) (Zhou et al., 2006), Hypervolume (HV) (While et al., 2006), and enhanced inverted generational distance (IGD-NS) (Tian et al., 2016), and so on. Adopting indicators to estimate the fitness of solutions, indicator-based MOEAs need to spend more computational costs to calculate the indicators. Decomposition-based MOEAs convert the original MOP into a set of single-objective optimization problems according to aggregation or scalarization functions. The representative algorithms are MOEA/D: A multi-objective evolutionary algorithm based on decomposition (MOEA/D) (Zhang and Li, 2007) and different variants of MOEA/D, such as based on MOEA/D with correlative selection mechanism (MOEA/D-CSM) (Liu R. et al., 2021), MOEA/D with adaptive weight vector adjustment (MOEA/D-AWA) (Qi et al., 2014), a scheme to use both differential evolution (DE) and covariance matrix adaptation in the MOEA/D (MOEA/D-CMA) (Li et al., 2016), and so on. The performances of the decomposition-based MOEAs are affected by the reference points or weight vectors adopted in the algorithms directly. For example, a set of uniformly distributed weight vectors may cause MOEA/D to perform unsatisfactorily when dealing with MOPs with irregular Pareto fronts.

#### 2.3.2. Framework of DK-MOEA

**Figure 4:**
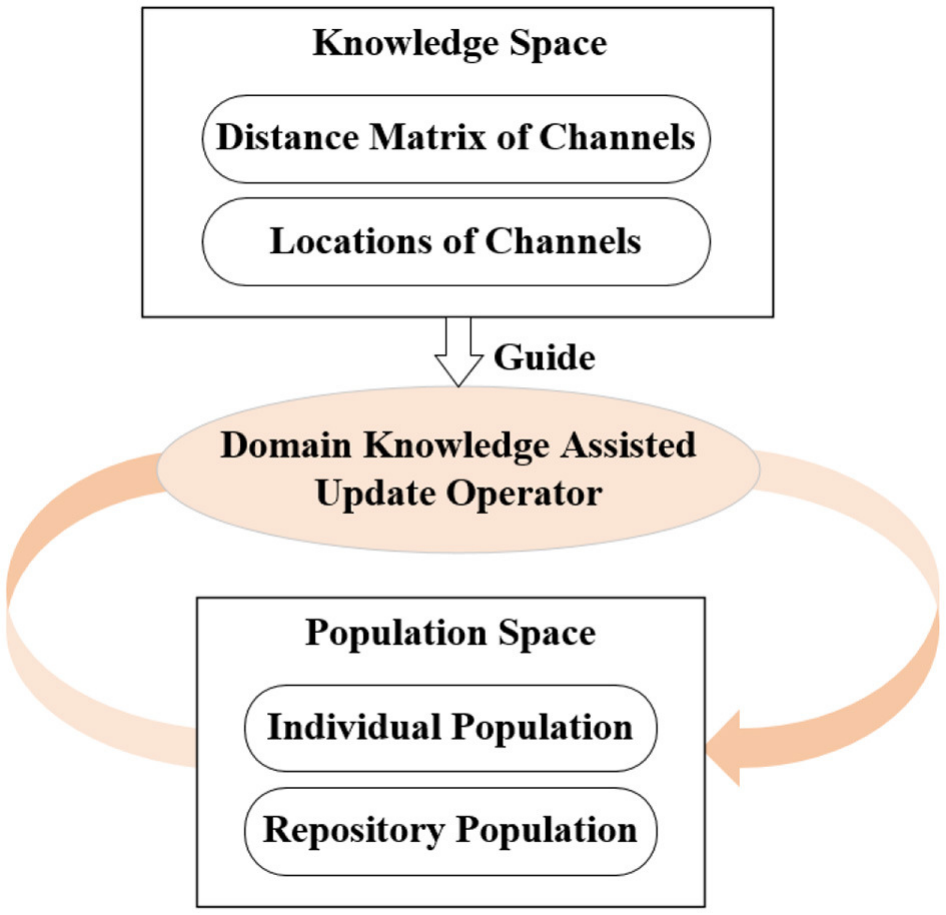
Framework of DK-MOEA.

In this paper, a domain knowledge-assisted multi-objective evolutionary algorithm (DK-MOEA) is proposed to solve the channel selection problem in BCIs. The framework of the proposed DK-MOEA is shown in Figure 4. In Figure 4, DK-MOEA has two spaces: the population space (*P*_*space*_) and the knowledge space (*K*_*space*_). In population space, the individual population (POP) contains a set of candidate solutions, while the repository space (REP) stores the non-dominated solutions found by DK-MOEA in the evolution process. The knowledge space in DK-MOEA contains domain knowledge, including the physical distance between channels and the location of the channels.

When the brain executes a specific task, not all channels provide signals that are valid for that task. If the correlation coefficient between a channel and all the other channels is 0, then this channel may provide redundant information. Studies have shown that the correlation between channels is related to their locations and the distance from each other (van den Broek et al., 1998). Therefore, the problem domain-related knowledge stored in the knowledge space is adopted to guide the evolution process of the population space in DK-MOEA.

The detailed procedure of DK-MOEA is given in Algorithm 2. Where *POP*_*t*_ and *REP*_*t*_, respectively, are the individual population and repository population in *P*_*space*_ at the *t*^*th*^ generation. In DK-MOEA, the tournament selection method (Osuna and Sudholt, 2022) and Simulated binary crossover (SBX) (Deb and Beyer, 2001) are adopted as the selection and crossover operators respectively. The detailed description of the two above-mentioned operators is not given for brevity, since they are widely adopted in a large number of MOEAs. It can be observed from Algorithm 2, DK-MOEA has two key steps: the initialization of the population and knowledge spaces (Line 1 in Algorithm 2) and the generation of offspring individuals by domain knowledge-assisted update operator (Line 8 in Algorithm 2). The detailed description of the two key steps presented in the next subsection.

**Algorithm 2:**
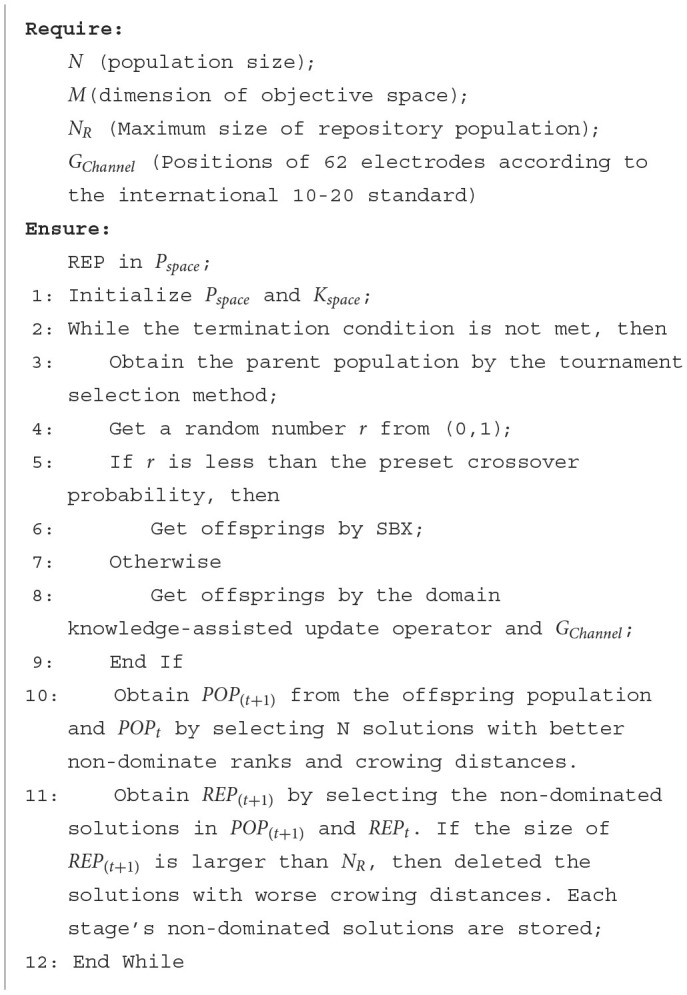
The detailed procedure of DK-MOEA.

#### 2.3.3. Initialization

**Figure 5:**
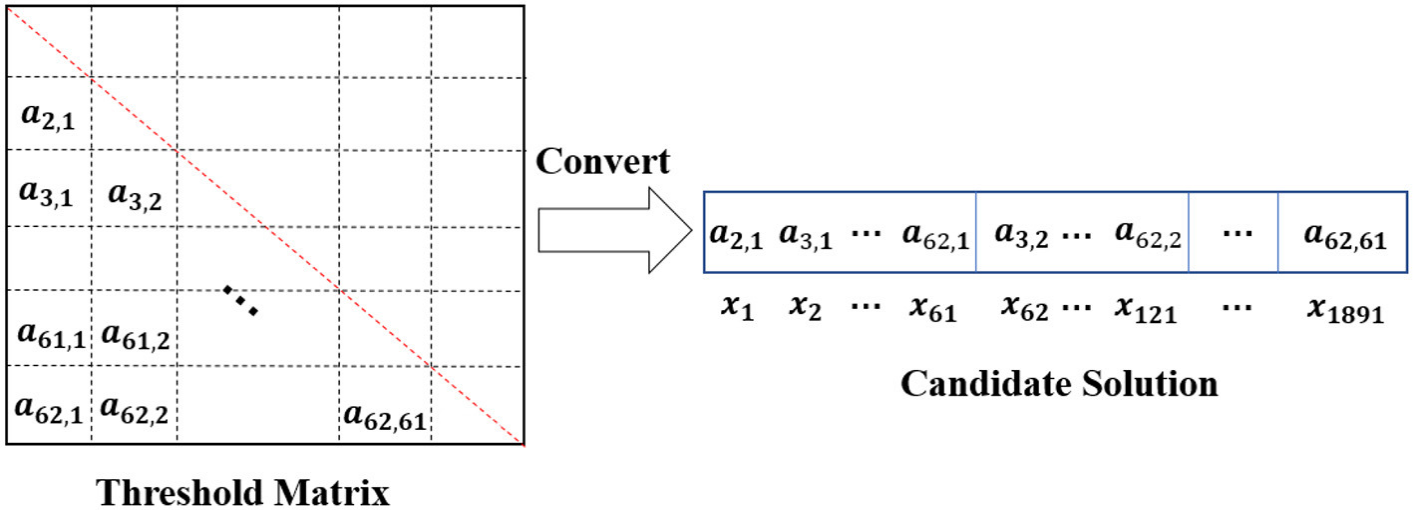
Illustration of a candidate solution.

As mentioned in Section 2.2, algorithms solve the channel selection problem by optimizing the threshold matrix. Therefore, POP contains *N* candidate threshold matrices, which have the same size as the EEG connective matrix. Since this paper adopts a 62-channel system, each individual in *POP* has their own 62 × 62 threshold matrix, whose elements take values between [−1,1]. The threshold matrix is utilized to filter the EEG connective matrices to determine whether one channel is linearly uncorrelated with the other channels. It is generally known that the EEG connective matrix is symmetric. As shown in Figure 2, both *c*_2, 1_ and *c*_1, 2_ indicate the correlation coefficient between channel 1 and channel 2. Therefore, only the threshold values of the lower or upper triangular part are needed to filter the EEG connective matrix. Furthermore, the threshold values on the diagonal are also not needed, as the filtering process needs to determine whether the two different channels are linearly independent. As shown in Figure 5, this section adopts the lower triangular part of a 62 × 62 threshold matrix and then converts the lower triangular part into a long vector as the decision variable *X* = {*x*_1_, *x*_2_, …, *x*_1891_}, which has 1,891 elements. In this case, *POP* can be initialized as a *N*×1891 matrix, in which each row is a candidate solution and each element takes a value generated from [−1,1] randomly. For each candidate solution in *POP*, every solution must process the connectivity matrices *D* = {*D*_1_, …, *D*_*n*_}, the objective values can be calculated for themselves as shown in Equation (4).


(7) $$\begin{array}{l} DM\left(k,l\right)=\sqrt{\overset{V}{\underset{sdv=1}{\sum }}{\left(G_{Channel_{k}}.sdv-G_{Channel_{l}}.sdv\right)}^{2}} \end{array}$$

For *K*_*space*_, the distance matrix of channels, denoted as *DM*, can be initialized by calculating the Euclidean distance between channels according to the corresponding position data information of the channels in the Figure 1. *G*_*channel*_, which is given in Additional Information to save space, stores information about the locations of channels in the cerebral cortex. In Equation (7), *G*_*channel*_.*sdv* means the spatial dimension value, i.e., the spatial coordinates, of the *k*^*th*^ Channel. The detailed information on *DM* is shown in Equation (7), which demonstrates that the Euclidean distance between channels ranges from 0 to 2. Where *V* is the spatial dimension of channels on the brain, and *sdv* is the value of the corresponding spatial dimension. In this paper, the spatial location of the channel is two-dimensional, and their corresponding position relationship is shown in Figure 1. Moreover, this paper introduces a location index to indicate the location of each channel, i.e., which hemisphere of the brain the channel is located in. *Location*_*i*_ = 1 and *Location*_*i*_ = −1 represent that the *i*^*th*^ channel is located in the left and right hemispheres of the brain, respectively. Furthermore, if the *k*^*th*^ channel and the *l*^*th*^ channel locate in the junction area between the left and right hemispheres of the brain, then the two channels are considered to be in the same hemisphere, i.e., *Location*_*k*_ = *Location*_*l*_.

#### 2.3.4. Domain knowledge-assisted update operator

In DK-MOEA, a domain knowledge-assisted update operator is proposed to generate offspring individuals. Research has shown that the EEG signals recorded from adjacent brain regions tend to be similar due to the volume conduction effect in the brain (van den Broek et al., 1998). Moreover, the asymmetry of the left and right hemispheres of the brain is closely related to the valence process of emotion (Coan and Allen, 2004; Reznik and Allen, 2018). Therefore, the domain knowledge, i.e., the distances between channels and the locations of channels, are adopted to help to improve the search efficiency in this paper. As shown in Algorithm 3, *X* = (*x*_1_, *x*_2_, …, *x*_γ_) is the individual's decision variables that need to be updated and γ is the dimension of the search space, which is 1,891 as described in Section 2.3.3. In Algorithm 3, α is the step factor (Lines 5 and 7), which controls the step size. The convergence time will be prolonged if α is too small. If α is set too high, the optimal solution may be missed, and it takes the value of 0.15 empirically. It can be observed that the update of *X* = (*x*_1_, *x*_2_, …, *x*_γ_) significantly depends on the parameter *Cr* (Lines 4–9 in Algorithm 3), which can be calculated according to the domain knowledge in *K*_*space*_.


(8) $$\begin{array}{l} Cr\left(k,l\right)=\frac{DM\left(k,l\right)-R}{2\left(Max\left(DM\right)+R\right)} \end{array}$$

**Algorithm 3:**
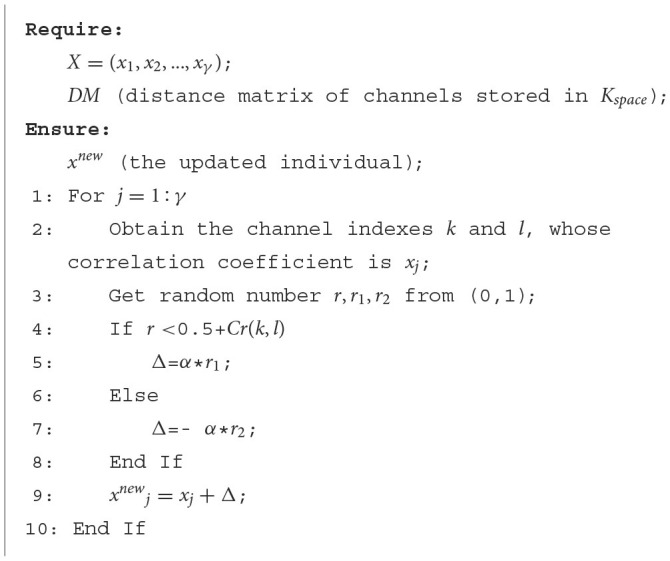
The detailed procedure of domain knowledge-assisted update operator.

In this section, two ways to get the value of *Cr* are presented. The first way only uses the distance information of channels, while the second way utilizes both distance and location information. The first and second ways to obtain *Cr* are given in Equations (8, 9), respectively. In Equations (8, 9), *R* is the distance radius of channels. *Max*(*DM*) is the maximum values in *DM*, which take the values of 2 according to the description in Section 2.3.3. If the *DM*(*k, l*) between channel *k* and channel *l* is less than *R*, it indicates the EEG signals obtained by the two channels are likely to be correlated in this case, the corresponding dimension in *X* = (*x*_1_, *x*_2_, …, *x*_γ_) tends to take a smaller value (Lines 7 and 9 in Algorithm 3). Conversely, *DM*(*k, l*)≥*R* indicates the EEG signals obtained by channel *k* and channel *l* are likely to be uncorrelated. Therefore, the corresponding dimension in *X* = (*x*_1_, *x*_2_, …, *x*_γ_) tends to take a larger value (Lines 5 and 9 in Algorithm 3) to make the correlation coefficient between channel *k* and channel *l* more likely to be 0 after filtering by the threshold matrix, the threshold matrix and *X* can convert each other, according to Figure 5. It can be observed from Equation (9), the second way to get *Cr* takes into consideration both the distance and location information of channels. If channel *k* and channel *l* locate in the same cerebral hemisphere, i.e., *Location*_*k*_ = *Location*_*l*_, then the calculation of *Cr* is the same as that in the first way as shown in Equation (8). If *Location*_*k*_ ≠ *Location*_*l*_, *Cr* will always take positive values in order to maintain a treatment to take a large value for the corresponding dimension in *X*. *Cr* in the case of *DM*(*k, l*) < *R* is less than that in the case of *DM*(*k, l*) ≥ *R*, since the latter's signals, obtained by two channels, are more likely to be uncorrelated when the distance between the two channels is larger than the distance radius.


(9) $$Cr(k,l)=\{\begin{array}{ll} \frac{DM(k,l)-R}{2(Max(DM)+R)} & if\;Location_{k}=Location_{l}\\ \frac{DM(k,l)}{2(Max(DM)+R)} & if\;Location_{k}\neq Location_{l}\\ & and\;DM(k,l)<R\\ \frac{DM(k,l)+R}{2(Max(DM)+R)} & if\;Location_{k}\neq Location_{l}\;and\\ & DM(k,l)\geq R \end{array}$$

## 3. Results

### 3.1. Experiment settings

To evaluate the performance of the proposed algorithm, DK-MOEA is compared to four well-performing and widely utilized algorithms, namely MOPSO (Coello and Lechuga, 2002), DEMO (Robič and Filipič, 2005), BIGA (Li et al., 2015), NSGA-II/SDR (Tian et al., 2018), and MOEA/D (Zhang and Li, 2007).

For a fair comparison, all algorithms have the same termination conditions and population sizes, i.e., the maximum number of function evaluations is set to 30,000, and the population size *N* is set to 200 for all algorithms. To ensure that ideal experimental results can be obtained, the parameter settings of each compared algorithm refer to the corresponding references. The parameter settings and references are shown in Table 1.

**Table 1:** Parameter settings for algorithms.

**Algorithm**	**Parameter setting**	**References**
MOPSO	Inertia weight *w* is 1; variation factor is 0.5; learning factor *C*_1_ and *C*_2_ are set to 1.49445.	Shi and Eberhart, 1998; Clerc, 1999
DEMO	Scaling factor *F* is 1.2; crossover parameter is 0.7.	Storn and Price, 1997; Robič and Filipič, 2005
BIGA	Probabilities of crossover and mutation are set to 0.5.	Li et al., 2015
NSGA-II/SDR	Probabilities of crossover and mutation are set to 0.5.	Tian et al., 2018
MOEA/D	Neighbor size is 20; probabilities of crossover and mutation are set to 0.5.	Zhang and Li, 2007
DK-MOEA	Distance radius *R* is set to 0.2; probabilities of crossover and mutation are set to 0.5.	

In this paper, the Hypervolume (HV) (Zitzler and Thiele, 1999) is used to evaluate the performance of algorithms. HV measures both the convergence and diversity of an algorithm by calculating the area of the hypercube, which is constructed by a reference point and the non-dominated solutions found by the algorithm. The larger the value of HV, the better the performance of the evaluated algorithm.

### 3.2. Comparison of DK-MOEA and other MOEAs on multi-objective channel selection problem

Table 2 presents the statistical results of all 6 algorithms over 30 independent runs on the multi-objective channel selection problem proposed in Section 2.2 in terms of HV. In Table 2, the best average HV values are shown in bold. The symbol “+”, “-”, and “≈” indicate the performance of the compared algorithm proposed DK-MOEA is significantly better than, worse than, and similar to that of the proposed DK-MOEA according to Wilcoxon rank-sum test (Yaman et al., 2021) with a significance level of 5%, respectively.

**Table 2:** Statistical values of *HV* obtained by DK-MOEA and other MOEAs.

**Subject**	**MOPSO**	**DEMO**	**BIGA**	**NSGA-II/SDR**	**MOEA/D**	**DK-MOEA**
1	3.04E + 00 (8.84E - 01) -	3.90E + 00 (1.02E + 00) -	1.15E + 00 (7.15E - 01) -	1.52E - 01 (3.38E - 03) -	3.77E + 00 (1.45E - 02)	**3.98E** **+** **01 (5.51E** **+** **00)**
2	2.12E + 00 (1.08E + 00) -	1.10E + 00 (3.24E - 01) -	2.86E + 01 (1.55E + 01) -	2.20E - 01 (1.69E - 03) -	2.98E + 00 (2.43E - 03)	**1.25E** **+** **02 (4.10E** **+** **00)**
3	2.77E + 00 (1.30E + 00) -	1.90E + 00 (4.52E - 01) -	1.61E + 00 (1.95E + 00) -	1.57E - 01 (3.72E - 03) -	7.32E + 00 (4.21E - 01)	**1.34E** **+** **02 (4.68E** **+** **00)**
4	1.07E + 00 (3.20E - 01) -	2.75E + 00 (7.96E - 01) -	2.46E - 01 (9.10E - 02) -	3.56E - 02 (1.22E - 02) -	1.13E + 00 (3.54E - 01)	**1.07E** **+** **02 (1.92E** **+** **01)**
5	**3.51E** **+** **01 (3.04E** **+** **01)** **≈**	2.03E + 00 (4.18E - 01) -	7.12E + 00 (1.38E + 01) ≈	1.50E - 01 (2.42E - 03) -	2.02E + 00 (4.61E - 01)	2.67E + 01 (4.98E + 00)
6	5.08E + 00 (1.09E + 00) -	1.00E + 00 (3.47E - 01) -	**3.46E** **+** **01 (3.29E** **+** **00)** **≈**	2.24E - 01 (8.45E - 02) -	1.05E + 00 (3.74E - 01)	2.84E - 01 (1.48E + 01)
7	3.70E + 00 (2.47E - 01) -	1.81E + 00 (4.50E - 01) -	8.71E - 01 (3.69E - 01) -	1.59E - 01 (6.28E - 02) -	5.01E + 00 (2.62E - 01)	**3.25E** **+** **01 (6.63E** **+** **00)**
8	1.78E + 00 (2.22E + 00) -	2.97E + 00 (3.47E - 01) -	1.23E + 00 (5.90E - 01) -	1.50E - 01 (5.06E - 03) -	2.81E + 00 (3.41E - 01)	**1.54E** **+** **02 (4.15E** **+** **00)**
9	9.08E - 01 (4.19E - 01) -	9.87E - 01 (2.29E - 01) -	5.70E + 00 (9.94E + 00) -	1.42E - 01 (4.09E - 03) -	7.42E - 01 (3.53E - 01)	**1.02E** **+** **02 (9.67E** **+** **00)**
+/-/≈	0/8/1	0/9/0	0/7/2	0/9/0	0/9/0	

As Table 2 shows, DK-MOEA obtains the best HV results for 7 out of 9 subjects. The main difference between the proposed DK-MOEA and the other comparative algorithms is that DK-MOEA adopts a two-space framework, in which domain-related knowledge is utilized to guide the evolution process. Therefore, the statistical results in Table 2 indicate the effectiveness of the proposed two-space framework. NSGA-II/SDR performs worse than the other algorithms relatively. This may be because NSGA-II/SDR adopts a strengthened dominance relation (SDR), which tends to choose solutions with better convergence properties, to get the next individual population. In this case, the non-dominated solutions found by NSGA-II/SDR are likely to concentrate on partial areas of the true Pareto fronts. For Subject 5 and Subject 6, MOPSO and BIGA achieve the best results in terms of HV, respectively. DK-MOEA obtains the second-best HV results for both Subject 5 and Subject 6. In MOPSO, the personal and global best positions can help the algorithm strike a balance between exploration and exploitation. In BIGA, the bi-goal evolution strategy, which considers both proximity and diversity, helps the algorithm get the best performance for some subjects in the channel selection problem. MOEA/D decomposes the original multi-objective optimization problem into a set of simple single-objective problems to obtain a Pareto-optimal set with better distribution. However, the channel selection problem employed in this paper is a large-scale multi-objective optimization problem with 1,891 decision variables. Without using any prior knowledge, it is easy to make the algorithm fall into local optima. The experimental results in Table 2 show that the MOEA/D does not show significant advantages for the channel selection problems.

**Figure 6:**
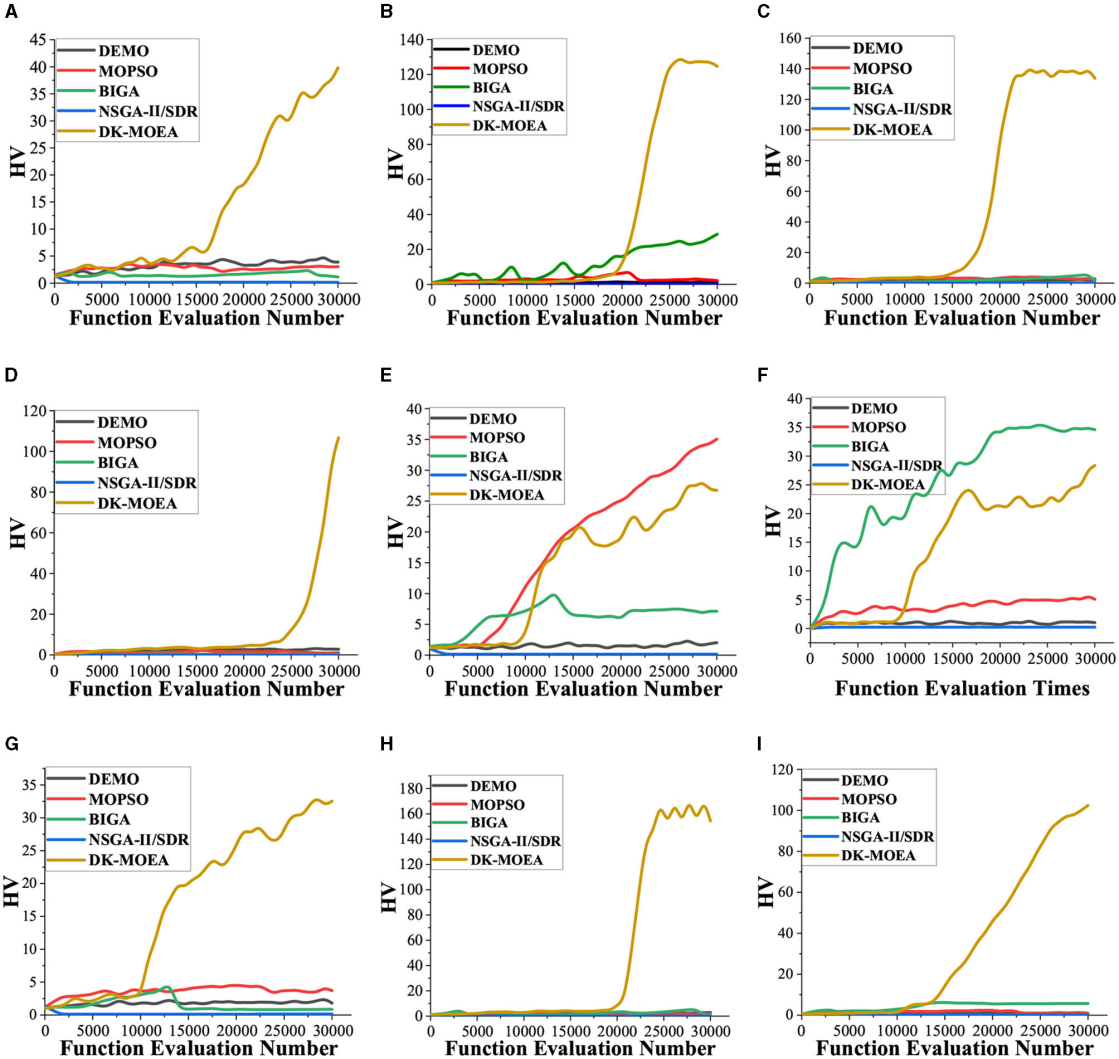
Convergence of 5 algorithms on 9 subjects. **(A)** Subject 1. **(B)** Subject 2. **(C)** Subject 3. **(D)** Subject 4. **(E)** Subject 5. **(F)** Subject 6. **(G)** Subject 7. **(H)** Subject 8. **(I)** Subject 9.

Figure 6 demonstrates the average HV value obtained by all 6 comparative algorithms as the function evaluation number increases. It can be observed from Figure 6, DK-MOEA gets good HV results faster than the other algorithms for most subjects. Therefore, the results in Figure 6 verify the effectiveness of the proposed two-space frameworks and indicate that utilizing the useful knowledge extracted from the problem domain can enhance the search efficiency of the algorithm.

**Figure 7:**
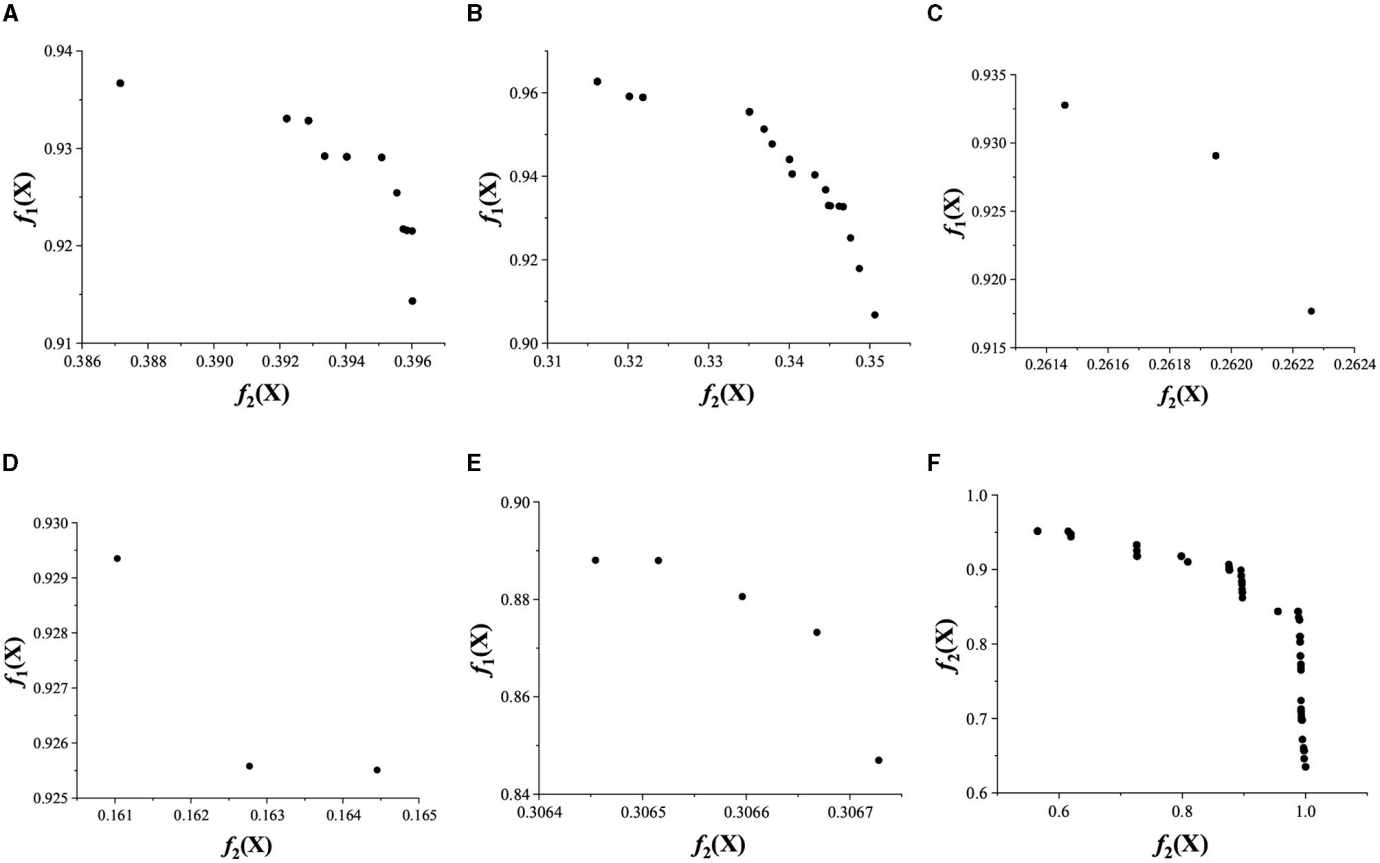
Pareto fronts of all algorithms on subject 2. **(A)** MOPSO. **(B)** DEMO. **(C)** BIGA. **(D)** NSGA-II/SDR. **(E)** MOEA/D. **(F)** DK-MOEA.

To better display the comparison results, the Pareto fronts of DK-MOEA and all other compared algorithms are given in Figure 7. In Figure 7, *f*_1_(*X*) and *f*_2_(*X*) are the two objectives, which has been described in Section 2.2, of the obtained Pareto-optimal solutions (denoted as *X*). Specifically, *f*_1_(*X*) is the classification accuracy, and *f*_2_(*X*) indicates the degree of channel deletion of *X*. It can be observed from Figure 7 that some algorithms, i.e., BIGA, NSGA-II/SDR, and MOEA/D, obtain relatively few solutions on the Pareto front. This means that the above two algorithms have relatively weak search capabilities. MOPSO, DEMO, and DK-MOEA have obtained more solutions on the Pareto front. However, the objective values of MOPSO and DEMO concentrate in a small range, which means MOPSO and DEMO is easily trapped into local optima. The Pareto-optimal solutions obtained by DK-MOEA show a good distribution on the Pareto front, which means that the proposed algorithm performs better in solving channel selection problems.

### 3.3. Comparison of DK-MOEA and traditional classification algorithm with all channels

**Figure 8:**
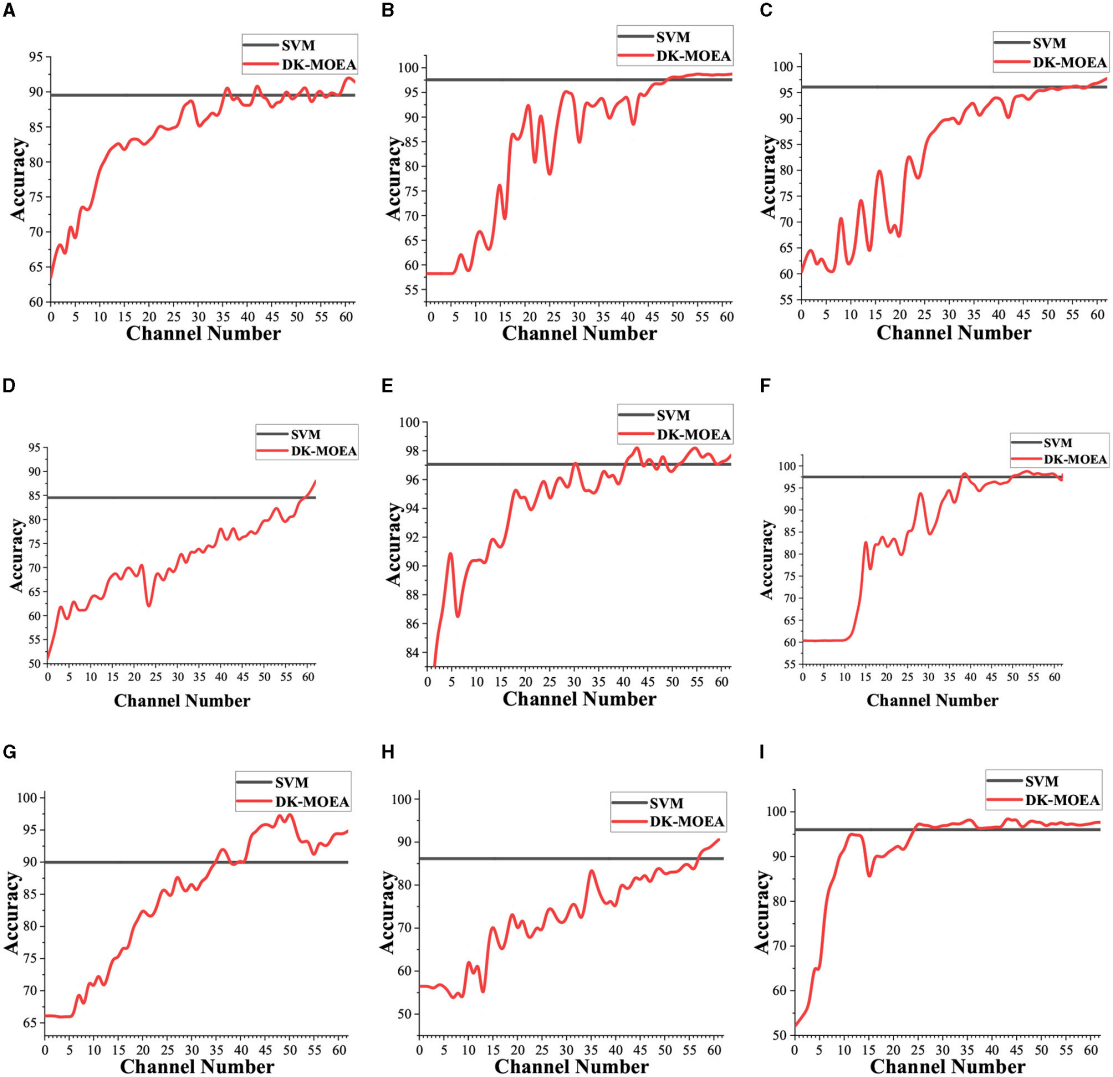
Convergence of DK-MOEA and SVM on 9 subjects. **(A)** Subject 1. **(B)** Subject 2. **(C)** Subject 3. **(D)** Subject 4. **(E)** Subject 5. **(F)** Subject 6. **(G)** Subject 7. **(H)** Subject 8. **(I)** Subject 9.

In this section, the traditional algorithm, which uses all channels rather than selecting a part of channels, is adopted. Similar to DK-MOEA and the other MOEAs in Section 3.2, the traditional algorithm chooses SVM as the classifier and adopts connectivity matrices as the input feature. In Figure 8, the red lines demonstrate the average classification accuracy with different numbers of channels in the Pareto-optimal solution sets obtained by DK-MOEA over 30 independent runs. The black lines give the classification accuracy obtained by SVM adopting all 62 channels. As Figure 8 shows, with the increase in the number of selected channels, the classification accuracy achieved by DK-MOEA also increases. It also can be observed from Figure 8, the classification accuracy of DK-MOEA with a part of channels is better than that of SVM with all channels. For example, the classification accuracy of DK-MOEA with 36 channels is better than that of SVM with all 62 channels. This phenomenon reveals the significance of this work, which tries to use as few channels as possible without reducing the classification accuracy. With all 62 channels, DK-MOEA gets better results than SVM for most subjects. This is because the connectivity matrix adopted in DK-MOEA has been filtered and reduced some redundant information compared to the original connectivity matrix. Therefore, the results in Figure 8 demonstrate the EEG signals collected from all 62 channels contain redundant information, which may cause the degradation of the classification accuracy.

## 4. Discussion

### 4.1. Investigation of the distance radius *R*

**Figure 9:**
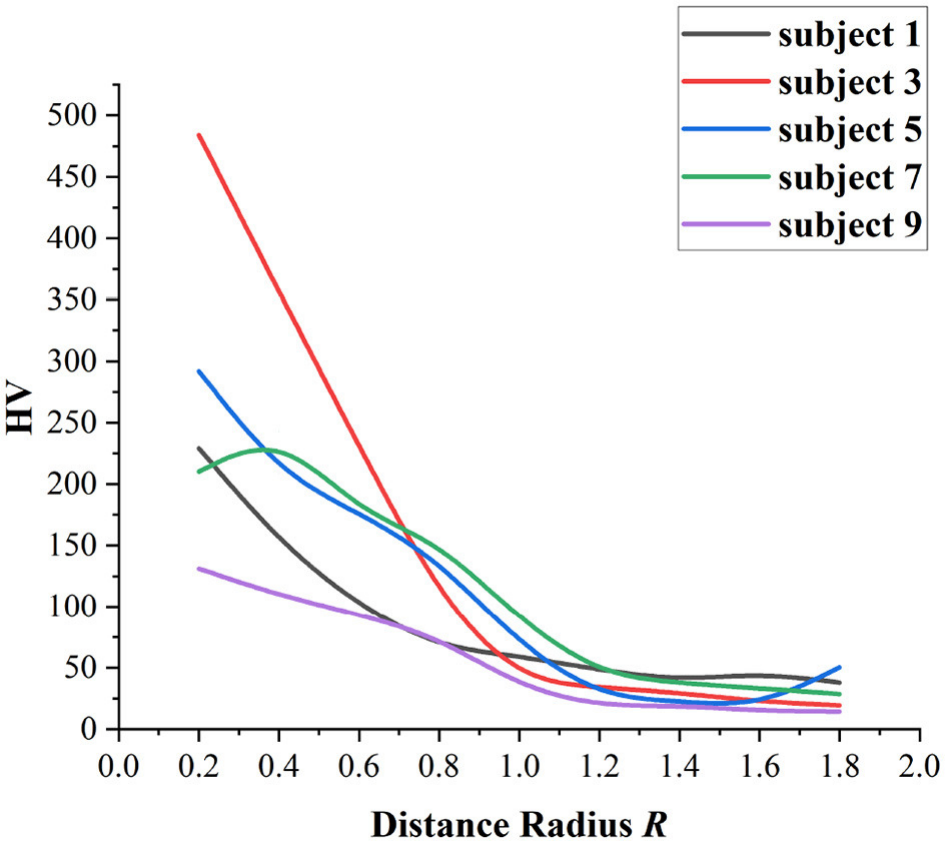
Investigation of the distance radius R.

In DK-MOEA, the distance radius *R* plays an important role in the proposed domain knowledge-assisted update operator. If the distance between two channels is larger than *R*, then the two channels will tend to be regarded as uncorrelated, as shown in Algorithm 1. Therefore, if *R* is too large, then too many channels will be considered unrelated to each other. In this case, too many channels may be deleted and thus resulting in the reduction of the classification accuracy. In this section, 5 subjects, including subject 1, subject 3, subject 5, subject 7, and subject 9, are selected to investigate the influence of different *R* values on the channel selection problem formulated in Section 3. Figure 9 gives the average HV values of DK-MOEA with different *R* over 30 independent runs. *R* ranges from 0.2 to 1.8 since the maximum distance between two channels is 2. It can be observed from Figure 9, *R* = 0.2 achieves the best performance for most of the tested subjects. For subject 7, DK-MOEA gets the best and second-best HV values when *R* is set to 0.3 and 0.2, respectively. Therefore, the distance radius *R* is set to 0.2 in this paper.

### 4.2. Investigation of the domain knowledge assisted update operator

**Figure 10:**
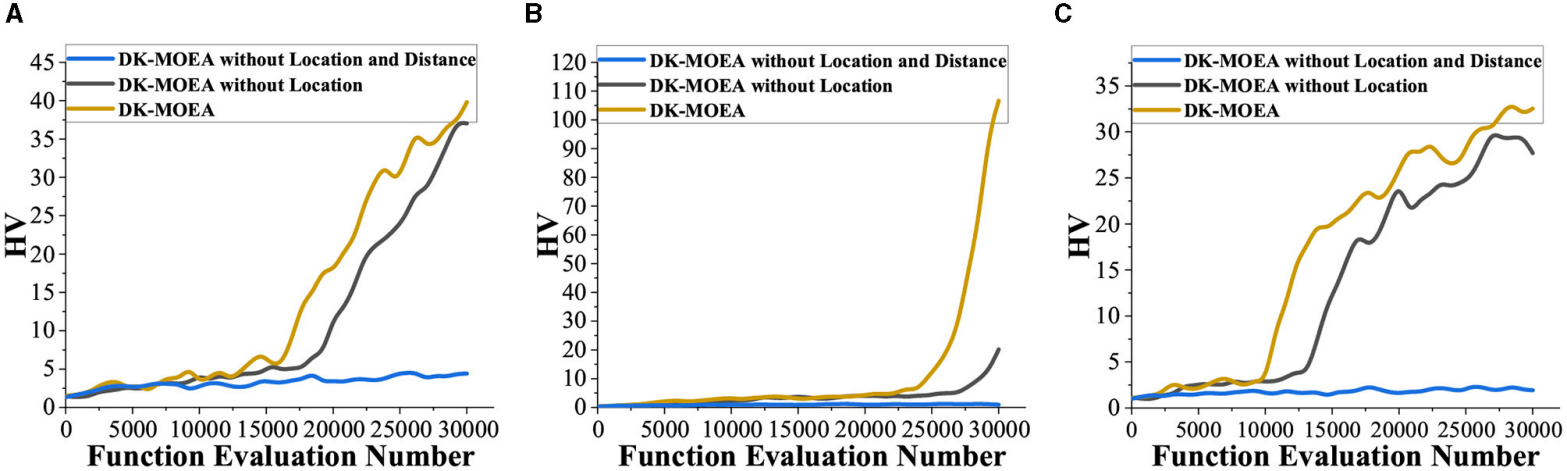
Investigation of the effectiveness of the domain knowledge-assisted update operator. **(A)** Subject 1. **(B)** Subject 4. **(C)** Subject 7.

In DK-MOEA, the key operator is the domain knowledge-assisted update operator, which improves the search efficiency of DK-MOEA by utilizing domain-related knowledge, namely the locations of channels and the distance matrix between channels. In this section, Subject 1, Subject 4, and Subject 7 are taken as examples and HV is used to evaluate the effectiveness of algorithms in terms of convergence and diversity. As shown in Figure 10, DK-MOEA without Location and Distance performs the worst for all three subjects, which means the location and distance information extracted from the problem domain helps enhance the ability to solve the channel selection problem. It can be observed from Figure 10, DK-MOEA achieves better performance and converges faster than DK-MOEA without Location. This indicates that, in addition to the location information of channels, the distance matrix between channels also provides great help for channel selection problems.

## 5. Conclusions and future work

In this paper, the channel selection problem in BCIs is formulated as a two-objective optimization problem to achieve the balance between number of channels and classification accuracy. After that, a domain knowledge-assisted multi-objective evolutionary algorithm (DK-MOEA) is proposed to solve the formulated multi-objective channel selection problem. DK-MOEA adopts a two-space framework, which contains two spaces, namely population space, and knowledge space. The knowledge space stores the locations of channels and the distance matrix between channels, which can be adopted to guide the evolution process of DK-MOEA. The proposed algorithm has been evaluated on a fatigue detection task with 9 volunteers and compared with 4 state-of-the-art MOEAs. The experimental results demonstrate the proposed algorithm can achieve better performance for the fatigue detection task. This indicates that the knowledge extracted from the problem domain can improve the performance of the algorithm. Moreover, the comparison of DK-MOEA and SVM with All Channels demonstrates that a larger number of channels will not always lead to better classification results. Therefore, it is possible to select as few electrodes as possible without reducing the classification accuracy. This paper aims to make a balance between number of channels and the accuracy of the fatigue detection task, which can not only reduce the complexity of subsequent data processing but also make the practical application more convenient.

As shown in Section 2.3.3, each individual in DK-MOEA is a threshold matrix and the matrix will be converted to a long vector that has 1,891 members. So, the channel selection problem can be regarded as a large-scale multi-objective problem. Moreover, many elements in a candidate solution are set to 0 after filtering. In this case, the optimization problem in this paper can be further regarded as a sparse large-scale optimization problem. Therefore, how to combine the characteristics of sparse large-scale problems in the evolution process to improve the performance of the algorithm is one of the future works of this paper.

## Data availability statement

The raw data supporting the conclusions of this article will be made available by the authors, without undue reservation.

## Author contributions

TL and AY contributed to conception and design of the study. AY organized the database. TL performed the statistical analysis and wrote the first draft of the manuscript. All authors contributed to manuscript revision, read, and approved the submitted version.
