# Supplementary material for: Domain knowledge-assisted multi-objective evolutionary algorithm for channel selection in brain-computer interface systems
Source: Front Neurosci. 2023 Sep 7;17:1251968. doi: 10.3389/fnins.2023.1251968 (PMC10512944; doi:10.3389/fnins.2023.1251968)
Supplement: Supplementary file 1 [file Data_Sheet_1.PDF]

Detailed position information stored in $G_{channel}$.

No	Name	X	Y	No	Name	X	Y
1	Fp1	0.950477274216144	0.308828787214590	33	TP7	-0.308828787214590	0.950477274216144
2	Fp1	0.999390948835232	0	34	CP5	-0.338841924096412	0.882713391123370
3	Fp2	0.950477274216144	-0.308828787214590	35	CP3	-0.359638188621299	0.676381059289295
4	Fp2	0.884845170874598	0.375594491280951	36	CP1	-0.374713691632017	0.374713691632017
5	AF4	0.884845170874598	-0.375594491280951	37	CPz	-0.390737554815941	-4.78515495812006e-17
6	F7	0.587427260999931	0.808524261632206	38	CP2	-0.374713691632017	-0.374713691632017
7	F5	0.633706137784739	0.728995520369607	39	CP4	-0.359638188621299	-0.676381059289295
8	F3	0.673024075906577	0.545004150624971	40	CP6	-0.338841924096412	-0.882713391123370
9	F1	0.710268200264536	0.286966980283610	41	TP8	-0.308828787214590	-0.950477274216144
10	Fz	0.719349499529124	0	42	P7	-0.587427260999931	0.808524261632206
11	F2	0.710268200264536	-0.286966980283610	43	'P5	-0.633706137784738	0.728995520369608
12	F4	0.6730240759065	-0.545004150624	44	P3	-0.673024075906577	0.5450041506249
13	F6	0.633706137784739	-0.728995520369607	45	P1	-0.710268200264536	0.286966980283610
14	F8	0.587427260999931	-0.808524261632206	46	Pz	-0.719349499529124	-8.80949062066592e-17
15	FT7	0.308828787214590	0.950477274216144	47	P2	-0.710268200264536	-0.286966980283610
16	FC5	0.338841924096412	0.882713391123370	48	P4	-0.673024075906577	-0.545004150624971
17	FC3	0.359638188621300	0.676381059289294	49	P6	-0.633706137784738	-0.728995520369608
18	FC1	0.374713691632017	0.374713691632017	50	P8	-0.587427260999931	-0.808524261632206
19	FCz	0.390737554815941	0	51	PO7	-0.808524261632206	0.587427260999931
20	FC2	0.374713691632017	-0.374713691632017	52	PO5	-0.834536022940231	0.541954030094238
21	FC4	0.359638188621300	-0.676381059289294	53	PO3	-0.884845170874598	0.375594491280951
22	FC6	0.338841924096412	-0.882713391123370	54	POz	-0.933576673625621	-1.14330168511425e-16
23	FT8	0.308828787214590	-0.950477274216144	55	PO4	-0.884845170874598	-0.375594491280951
24	T7	6.11950463293952e-17	0.999390948835232	56	PO6	-0.834536022940231	-0.541954030094238
25	C5	5.71650842557125e-17	0.933576673625621	57	PO8	-0.808524261632206	-0.587427260999931
26	C3	4.40474531033296e-17	0.719349499529124	58	CB1	-0.541678393551062	0.541678393551062
27	C1	2.39257747906003e-17	0.390737554815941	59	O1	-0.950477274216144	0.308828787214590
28	Cz	3.74939945665464e-33	-6.12323399573677e-17	60	Oz	-0.999390948835232	-1.22390092658790e-16
29	C2	2.39257747906003e-17	-0.390737554815941	61	O2	-0.950477274216144	-0.308828787214590
30	C4	4.40474531033296e-17	-0.719349499529124	62	CB2	-0.541678393551062	-0.541678393551062
31	C6	5.71650842557125e-17	-0.390737554815941				
32	T8	6.11950463293952e-17	-0.999390948835232				
